# Demographic, clinical and tomographic characteristics of pellucid marginal degeneration patients in South Egyptian population

**DOI:** 10.1007/s10792-022-02326-1

**Published:** 2022-08-24

**Authors:** Amr Mounir, Marwa Mahmoud Abdellah, Islam Awny, Ahmed Hassan Aldghaimy, Engy Mohamed Mostafa

**Affiliations:** 1 Ophthalmology Department, Faculty of Medicine, Sohag University, Nasr City, Sohag 82524 Egypt; 2 Ophthalmology Department, Faculty of Medicine, South Valley University, Qena, Egypt

**Keywords:** Pellucid marginal degeneration, Tomographic characteristics, South Egyptian Population

## Abstract

**Purpose:**

To retrospectively evaluate the demographic, clinical, and tomographic characteristics of pellucid marginal degeneration (PMD) patients in South Egypt.

**Methods:**

This study was retrospective cross-sectional, including all patients who attended and sought refractive surgery at Sohag Center for Corneal and Refractive Surgeries, Sohag, South Egypt, between October 2016 and October 2020, and had a diagnosis of PMD. It included cases of PMD at different stages. Cases with PMD were diagnosed by the two authors (experienced in corneal refractive surgery), combining both slit-lamp biomicroscopy findings and corneal tomography.

**Results:**

Out of the 2534 patients attending the Sohag Center for Corneal and Refractive Surgeries (between October 2016 and May 2020) seeking correction of their refractive errors, 24 patients were found to fit in the diagnosis of PMD. Forty-three eyes of the 24 patients were diagnosed with PMD. The topographic patterns ranged from horizontal bow tie (against the rule astigmatism) being the least presenting, followed by crab claw, then butterfly pattern.

**Conclusion:**

PMD is a separate entity of the ectatic corneal spectrum that can easily be misinterpreted as Keratoconus. Topographic and tomographic patterns cannot solely diagnose PMD as they should be enforced by slit-lamp findings.

## Introduction

Pellucid marginal degeneration (PMD) is defined as a non-inflammatory, progressive peripheral ectatic corneal disease characterized by a narrow band of inferior corneal thinning separated from the limbus by an uninvolved area [1]. This ectatic corneal morphology causes against-the-rule astigmatism accompanied over time with visual loss [2].

The term “pellucid” means clear. It was used to describe the clarity of the cornea and the absence of any corneal scarring, lipid deposition, or corneal vascularization, despite the presence of ectasia [3].

Corneal topographic analysis shows flattening in the vertical meridian, inducing a marked against-the-rule astigmatism and a significant steepening around the area of maximum thinning [4]. This corneal pattern corresponds with a topographic map that shows the classical “butterfly” pattern [5].

The management of PMD includes various modalities of treatment, including spectacles, rigid gas-permeable contact lenses [6]. Surgical management includes intracorneal ring segments [7], full-thickness crescentic wedge resection [8], lamellar crescentic wedge resection [9], deep anterior lamellar keratoplasty [10], and penetrating keratoplasty (PK) [11].

Studying PMD cases and differentiating them from Keratoconus (KCN) cases is of pivotal importance in everyday refractive practice, given the fact that KCN has been extensively studied with little light shed on PMD. The main location of maximal corneal thinning and steepening are the main findings that clinically differentiate between both entities, with PMD corneal thinning being more peripheral. [12].

Our study aimed to retrospectively evaluate the demographic, clinical, and tomographic characteristics of PMD patients in South Egypt.

## Patients and methods

This study was retrospective cross-sectional, including all patients who attended and sought refractive surgery at Sohag Center for Corneal and Refractive Surgeries, Sohag, South Egypt, between October 2016 and October 2020, and had a diagnosis of PMD.

The patients’ records were evaluated. The data extracted included the demographic data (age, sex, and laterality) and clinical data (uncorrected visual acuity (UCVA), best corrected visual acuity (BCVA) in decimal notion, sphere, cylinder along with slit-lamp biomicroscopy findings. Corneal tomography data were evaluated using Sirius Scheimpflug Placido topography (CSO, Florence, Italy). Corneal tomography findings recorded were keratometry, pachymetry, elevation, and tomographic indices and patterns (exaggerated against-the-rule astigmatism, crab claw, and butterfly shape).

Inclusion criteria included cases of PMD at different stages. Cases with PMD were diagnosed by the two authors (experienced in corneal refractive surgery), combining both slit-lamp biomicroscopy findings and corneal tomography: On slit-lamp examination: clear thinned band in the inferior peripheral corneal zone separated from the limbus by a 1–2 mm clear zone.1: against the rule astigmatism with flattening of at least one diopter along a vertical or oblique axis and a crescent-shaped steepening in the inferior part of the cornea extending toward the line perpendicular to the axis of flattening; 2: crab claw pattern; 3: bell shape detected by the Sirus tomographer. Extra caution was taken to differentiate patients with a crab claw pattern from inferior KCN according to the slit-lamp examination. Exclusion criteria included previous ocular surgery, corneal scarring, or opacity. Eyes diagnosed as KCN suspects were excluded from the study.

### Statistical analyses

The mean difference and standard deviation were calculated for all variables using SPSS 22.0 for Windows (SPSS Inc., Chicago, IL, USA). Descriptive statistics were presented as mean ± SD, frequency distribution, and percentage.

## Results

Out of the 2534 patients attending the Sohag Center for Corneal and Refractive Surgeries (between October 2016 and May 2020) seeking correction of their refractive errors, 24 patients were found to fit in the diagnosis of PMD.

Forty-three eyes of the 24 patients were diagnosed with PMD. Males constituted 62.5% (15 patients) of the cases reported. The majority of patients (19 patients) presented with bilateral affection, and only four had a unilateral presentation. The mean age of presentation was 32.6 ± 7.1 (ranging from 29 to 50). The sphere component of refraction ranged from + 3.00 to − 15.00D, while cylindrical power ranged from − 2.00 to − 10.0D. The spherical equivalent was myopic in all cases. Demographic and clinical data are represented in Table 1.

**Table 1:** Demographic and clinical data of PMD eyes (*n* = 43)

	Mean ± SD
Age (years)	32.6 ± 7.1
Sex (M/F)	(15/9)
UCDVA	0.08 ± 0.04
BCDVA	0.3 ± 0.08
Sphere (D)	− 2.0 ± 3.41
Cylinder (D)	− 4.56 ± 1.78
K flat (D)	41.8 ± 2.3
K steep(D)	46 ± 2.5
K max(D)	49.13 ± 5.54
Thinnest pachymetry (µm)	493 ± 52.5
Anterior corneal elevation (µm)	30.07 ± 26.5
Posterior corneal elevation (µm)	48.8 ± 46.3

*PMD* pellucid marginal degeneration, *UCDVA* uncorrected distance visual acuity, *BCDVA* best corrected distance visual acuity, *K* Keratometry

**Fig. 1:**
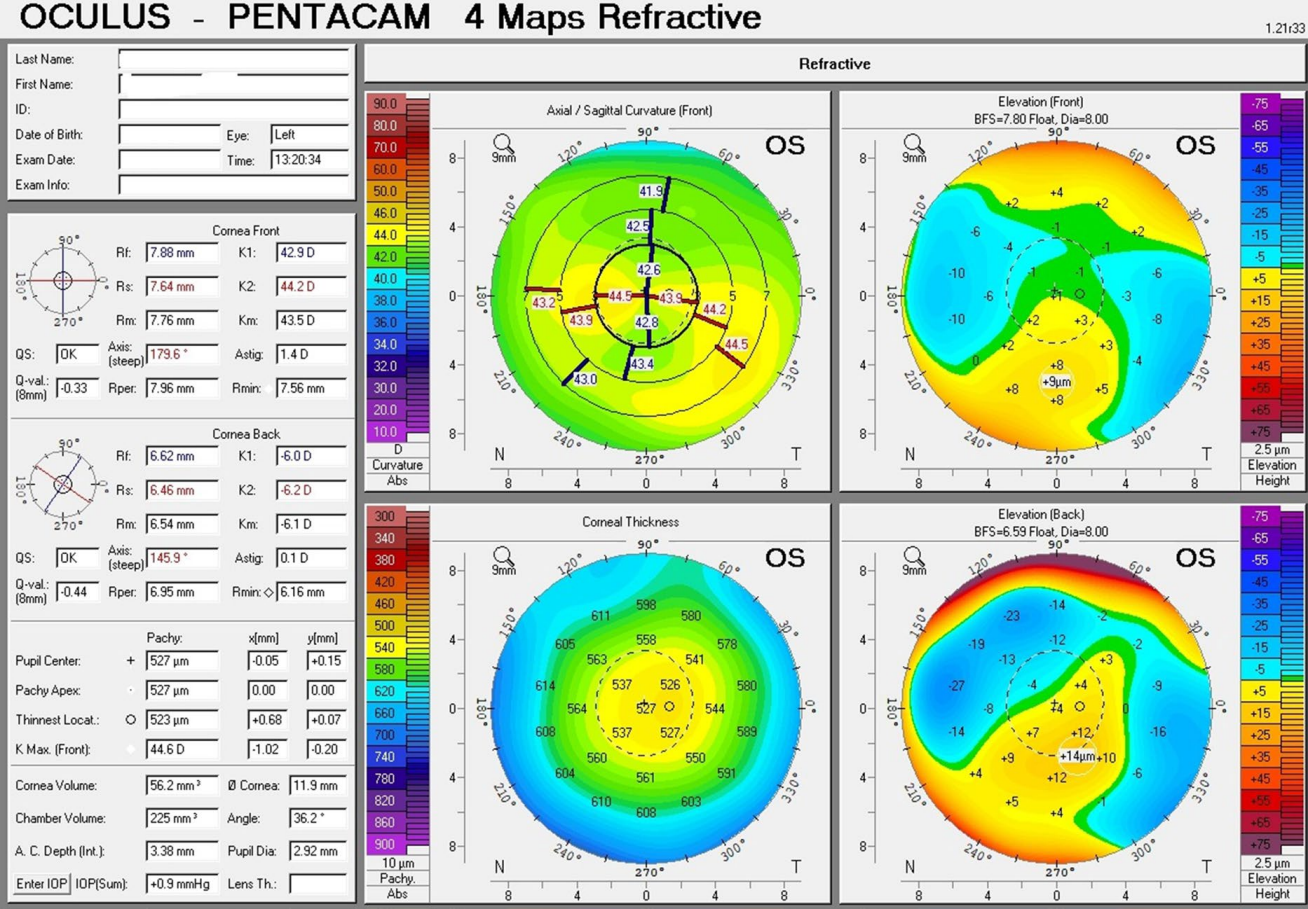
A printout of corneal topography of a case of Pellucid Marginal Degeneration with horizontal bow tie (against the rule astigmatism)

**Fig. 2:**
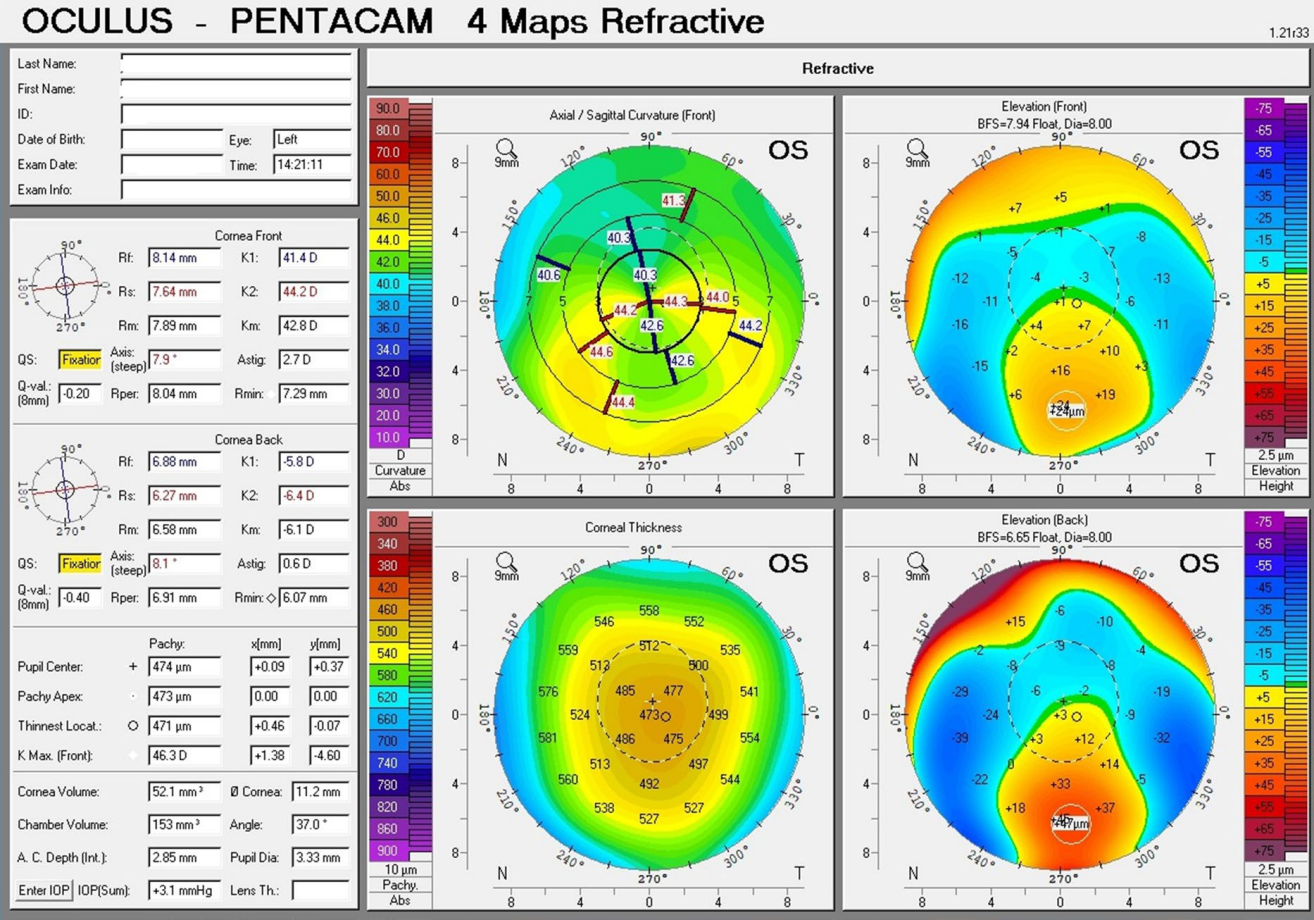
A printout of corneal topography of a case of Pellucid Marginal Degeneration with crab claw sign

**Fig. 3:**
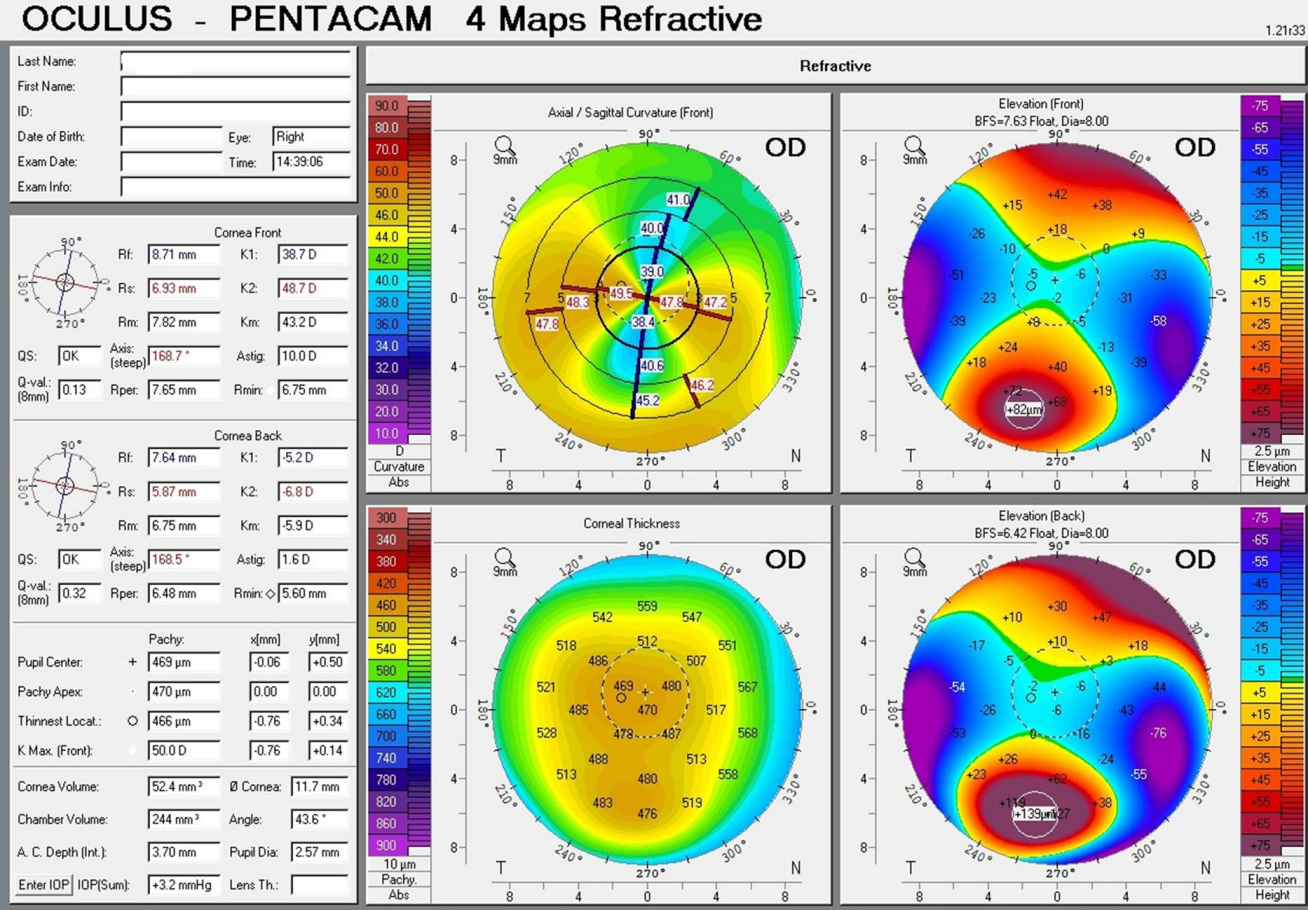
A printout of corneal topography of a case of Pellucid Marginal Degeneration with the butterfly pattern

The topographic patterns (shown in Table 2) ranged from horizontal bow tie (against the rule astigmatism) (Fig. 1) being the least presenting; followed by crab claw (Fig. 2), then butterfly pattern (Fig. 3).

**Table 2:** Corneal topographic characteristics in PMD eyes (*n* = 43)

Tomographic pattern	
Butterfly	20
Crab claw	18
Horizontal bow tie	5

Table 3 shows the pattern of distribution of Kmax position and corneal thinnest point location, as they did not co-apt to each other.

**Table 3:** Kmax position and corneal thinnest point location

K max location	
Below the inferior border of the pupil	20
Peripheral (near limbus)	23
Corneal thinnest point location	
Center	17
Inferior (near limbus)	26

## Discussion

Since PMD has a substantial impact on visual acuity and outcomes of refractive surgery, the detection of this ectatic corneal disorder is critically necessary [13, 14].

Distinguishing PMD from KCN is crucial as they both differ in prognosis and management [15, 16]. PMD is less aggressive with delayed intervention than KCN.

Bearing in mind that mixed forms of PMD and KCN do not exist [1], challenges arise in differentiating PMD from inferior KC. Yet, they can be overcome because PMD patients usually present with topographical signs decades later than KC [5].

Sridhar et al. [13], with the data of 116 eyes from 58 patients, noted an increased incidence of PMD in males (77.6%).

These findings agree with our results reported in males and other studies by Kompella et al. [6] (80%) and Tzelikis et al. [11] (66.7%). In contrast, no sex predilection was reported in studies by Krachmer et al. [17].

In our study, the age at the time of presentation ranged from 29 to 50 years. PMD topographic patterns in our case series were predominantly divided between butterfly and crab claw patterns. Only five eyes showed against the rule of astigmatism, denoting early presentation. PMD diagnosis in its early stages is considered a challenge as it may present with against-the-rule-astigmatism (ATR) or subtle variation from atypical KCN or normal corneas displaying harmless ATR astigmatism. In the current study, ATR was at a relatively young age yet with no statistical data.

It should be taken into consideration that PMD needs to be differentiated from inflammatory peripheral corneal disorders as Terrien’s marginal degeneration, Mooren’s ulcer, and ulcers resulting from connective tissue disorders. Moreover, PMD needs further research to detect subclinical cases, e.g., KCN.

## Conclusion

PMD is a separate entity of the ectatic corneal spectrum that can easily be misinterpreted as KCN. Topographic and tomographic patterns cannot solely diagnose PMD as they should be enforced by slit-lamp findings.

## Data Availability

This submission has not been published anywhere and is not simultaneously considered for any other publication.

## Declarations

### Research involving human and/or animal participants

This article does not contain any studies with animals performed by any of the authors.
